# Cyclizations Producing Hydrindanones with Two Methyl Groups at the Juncture Positions Mediated by Samarium Diiodide and Electrolysis

**DOI:** 10.3390/molecules170911079

**Published:** 2012-09-13

**Authors:** Masakazu Sono, Natsuko Ise, Tsutomu Shoji, Motoo Tori

**Affiliations:** Faculty of Pharmaceutical Sciences, Tokushima Bunri University, Yamashiro-cho, Tokushima 770-8514, Japan

**Keywords:** samarium diiodide, electrolysis, cyclization, hydrindanone

## Abstract

One-electron reductive intramolecular cyclization of enones with ketones or aldehydes mediated by samarium diiodide and electrolysis to afford *cis*-trimethyl- hydrindanolones. The reactions gave selectivities ranging from 1:1 to 100:0 depending on the conditions.

## 1. Introduction

**Figure 1:**
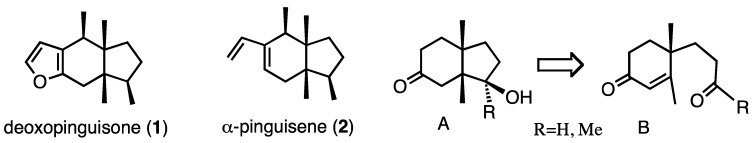
Target molecules and retrosynthesis.

Samarium diiodide has been used for many synthetic reactions [1,2,3,4] and the mechanism of its reactions has also been studied [5,6]. We are interested in preparing bicyclic compounds such as perhydroindenes [7,8], perhydronaphthalenes [9], and guaianes [10] and have published some results in this area. Compounds bearing methyl groups at the juncture positions are interesting from the synthetic point of view. For example deoxopinguisone (**1**) [11,12] and α-pinguisene (**2**) [13,14] (Figure 1) have four methyl groups in the bicyclo[4.3.0]nonane framework, all in a β-orientation; two of them are at the ring juncture positions, and have been synthetic targets [15,16]. We now planned to construct hydrindanone systems by 5-exo mode cyclization, although such a case involving a cascade cyclization giving one substituent at the ring-juncture position was described by Procter and his group [17,18]. Electrolysis is easy to carry out using water as a solvent, which is environmentally benign [19,20]. We now describe the carbon-carbon bond formation reactions leading from B to A mediated by SmI_2_ as well as electrolysis for the synthesis of hydrindanones (Figure 1).

## 2. Results and Discussion

**Scheme 1:**
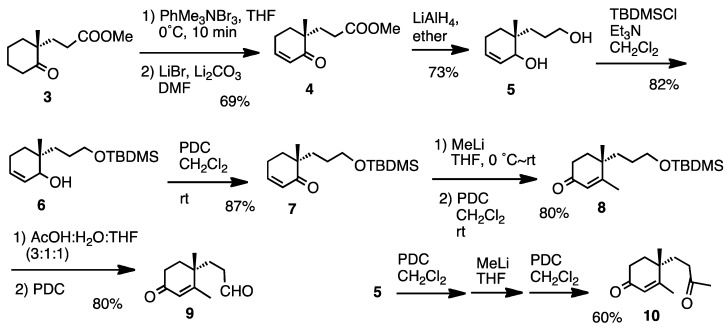
Preparation of compounds **9** and **10**.

For the synthetic work we chose compounds **9** and **10** (Scheme 1), which were prepared starting from ketone **3**. Several routine reactions afforded aldehyde **9** and ketone **10**. This route can also be used in chiral form, because compound **3** is now commercially available and easy to prepare [21].

Aldehyde **9** was subjected to reaction with SmI_2_ (3 equiv.) in THF with or without additives. The results are shown in Table 1. The products were isomeric keto-alcohols **11** and **12**, whose stereochemistries were established from the NOESY spectra. Without an additive, the reactions proceeded smoothly and both compounds were obtained in a ratio of 87:13 in favor of compound **11** at 0 °C (entry 1). When the reaction temperature was raised to rt (entry 2), the ratio of compound **12** increased to 31%. The yields were always good. When MeOH was added as a proton source, the ratio was about 7:3 in favor of compound **11** (entries 3 and 4). The yield varied from 47% to 58%. The reason why the yields were not so high is presumably due to the simple reduction of the double bond to give the corresponding dihydro derivatives, which were not isolated but detected in GC-MS. The ratio of **11** and **12** did not change very much when using HMPA (entries 7 and 8), however, when NiI_2_ was added (entries 9 and 10) the ratio of **12** was slightly increased [22].

**Table 1:** The reaction of aldehyde **9** with SmI_2_. 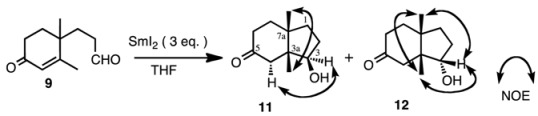

Entry	Additives	Temp (°C)	Yield (%)	Ratio	
				11	12
1	none	0	quant.	87	13
2	none	rt	quant.	69	31
3	MeOH 2 equiv.	0	58	72	28
4	MeOH 2 equiv.	rt	47	69	31
5	HMPA 12 equiv.	0	50	82	18
6	HMPA 12 equiv.	rt	44	84	16
7	NiI_2_	0	quant.	75	25
8	NiI_2_	rt	quant.	56	44

We next studied the reaction of ketone **10** under various conditions (Table 2). The products were isomeric keto-alcohols **13** and **14**, and a mixture of bicyclic compounds **15**, whose structures were determined by spectroscopic analyses. When ketone **10** was subjected to reaction with SmI_2_ without additive at 0 °C (entry 1), the products were **13** and **14** in a ratio of 84:16. In this case the major product had the hydroxy group in a β-orientation as determined by the NOESY spectrum (Table 2). 

**Table 2:** The reaction of compound **10** with SmI_2_. 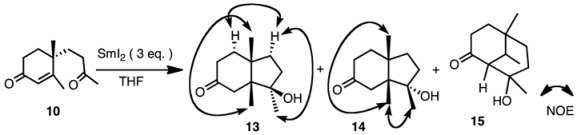

Entry	Additives	Temp. (°C)	Yield (%)	Ratio		
				13	14	15
1	none	0	quant.	84	16	-
2	none	rt	84	67	33	-
3	MeOH 2 equiv.	0	quant.	84	16	-
4	MeOH 2 equiv.	rt	quant.	55	45	-
5	HMPA 12 equiv.	0	78	61	17	22
6	HMPA 12 equiv.	rt	71	31	29	40
7	NiI_2_	0	quant.	53	47	-
8	NiI_2_	rt	85	86	14	-

When the temperature was raised to rt (entry 2), the ratio of compound **14** increased. This tendency was the same as that of the aldehyde mentioned above. However, when HMPA was added, a third product **15** was also obtained (entries 5 and 6). The ratio of **15** was 40% of the products at rt from a total yield of 71% (entry 6). This product was formed by an aldol type condensation of the samarium enolate of the α,β-unsaturated enone. When NiI_2_ was added, the yield was high and the ratio of compound **12** was slightly increased (entries 7 and 8) [22].

Electrolysis does not use organic solvents and expensive reagents, but rather water and electric power. In order to compare the selectivity, compounds **9** and **10** were subjected to electrolysis conditions as shown in Table 3. The yields were moderate and the ratio was **11**:**12** = 62:38 in the case of aldehyde **9**. The results were not very different from those of samarium iodide reduction. However, in the case of ketone **10**, only β-alcohol **13** was formed selectively (entry 2).

**Table 3:** The electrolysis of **9** and **10**. 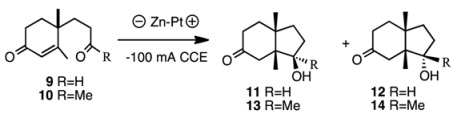

Entry	Solvent	Additive	Time (h)	SM	Yield (%)	Ratio
1	*^t^*BuOH-H_2_O (4:6)	Et_4_NTsO	2	**9**	53	**11:12** = 62:38
2	*^t^*BuOH-H_2_O (4:6)	Et_4_NTsO	2	**10**	76	**13:14** = 100:0

**Figure 2:**
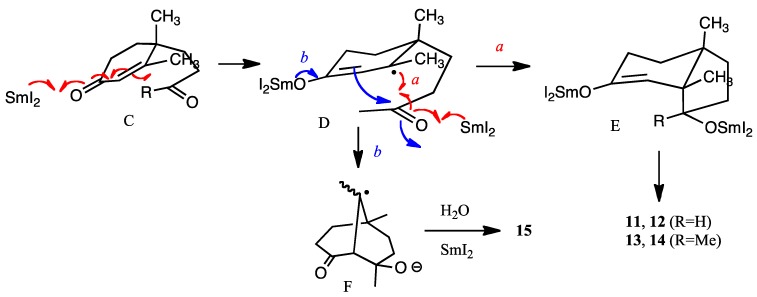
Mechanisms of reductive cyclization (R = H, Me).

Comparison of the half-wave potentials of α,β-unsaturated carbonyls with those of corresponding saturated carbonyl compounds has been extensively studied in electrochemistry [23]. The first waves of carbonyl groups, referred to as SCE., are −2.45 V (cyclohexanone), −2.25 V (methyl ethyl ketone), −1.8 V (propionaldehyde), −1.55 V (cyclohex-2-en-1-one), −1.50 V (acrolein), and −1.42 V (methyl vinyl ketone), respectively [23]. Therefore, the reduction of α,β-unsaturated carbonyl moiety seems easier than that of the isolated ketone carbonyl group with electrochemistry, but the selectivity of the one-electron reduction of the carbonyl moiety using SmI_2_ depends on the stereoelectronic properties of the substrate [6]. Samarium transfers one-electron to the carbonyl group of the enone moiety to form a radical anion (C to D) (Figure 2). Then one more samarium atom reduces the ketone carbonyl group and the carbon-carbon bond is formed as shown by arrow *a* to afford E (alkene first mechanism [2]). The hydroxy group is outside the bicyclic ring formed, because the samarium ion radical is large and the outside position is more energetically favored than inside of the ring. Thus, this configuration is more or less predominant. However, with HMPA as the additive, the reducing power must be higher than that with samarium alone [24], and the enone moiety is susceptible to reduction to afford a samarium enolate D. Then the aldol-type cyclization occurs to afford bicyclic anion radical F from D (shown by arrow *b* in Figure 2). Further reduction of this anion radical F and protonation afford product **15**. Electrolysis also creates a similar transition state leading to similar results. The reason why compound **10** produces **13** much more selectively is not clear at this stage. However, it is assumed that solvent molecules surround the methyl ketone carbonyl group resulting in the bulkier CO (solvent) moiety with consequent protrusion outside of the ring leading to the β-alcohol **13** [24].

## 3. Experimental

### 3.1. General

IR spectra were measured on a JASCO FT/IR-5300 spectrophotometer. The ^1^H and ^13^C-NMR spectra were taken using a Varian Unity 600 (at 600 MHz and 150 MHz, respectively) and a Varian Unity 200 (200 MHz and 50 MHz, respectively) spectrometer. Mass spectra including high-resolution mass spectra were recorded on a JEOL JMS-700 MStation. A Chemcopak Nucleosil 50-5 column (4.8 × 250 mm) was used for HPLC (JASCO pump system). For GC-MS an Agilent GC 6890 system equipped with a MS detector 5973 was used and the product ratios were determined by the area %. Silica gel 60 (70–230 mesh, Fuji Silysia) was used for column chromatography and silica gel 60 F_254_ plates (Merck) were used for TLC.

#### 3.1.1. General Procedure for Smi_2_ Reduction

A solution of substrate in dry THF was introduced into a solution of SmI_2_ at a certain temperature. Saturated solution of sodium potassium tartrate was added and the solvent was evaporated. The mixture was extracted with ether and worked up as usual. The residue was purified by silica-gel column chromatography.

#### 3.1.2. General Procedure for Electrolysis

Zn and Pt were used for the cathode and anode, respectively, with Et_4_NOTs in *t*BuOH-H_2_O (2:3) (20 mL), CCE at 100 mA (from −1.5 to −2.0 V *vs.* SCE) at rt. Work-up: benzene was added and most of the water was removed under reduced pressure. The residue was extracted with EtOAc and the organic layer was washed with Sat. NaCl solution. The organic layer was dried (MgSO_4_), and the filtrate was evaporated to give a residue, which was purified by silica-gel column chromatography or HPLC.

### 3.2. Preparation of Methyl 3-(1-Methyl-2-oxocyclohex-3-enyl)propanoate *(**4**)*

Ketone (**3**, 1 g, 0.5 mmol) was treated with phenyltrimethylammonium tribromide (1.9 g, 0.88 mmol) in THF (5 mL) at 0 °C for 10 min. After the usual work-up, the residue (925 mg) was successively treated with LiBr (585 mg, 5.6 mmol) and Li_2_CO_3_ (148 mg, 3.3 mmol) in DMF (4 mL) at 150 °C for 18 h. Usual work-up and purification afforded enone **4** (684 mg, 69%). Oil; IR (FT): 1730, 1660, 1630 cm^−1^; ^1^H-NMR (200 MHz, CDCl_3_): δ 1.09 (3H, s), 1.73–1.99 (4H, m), 2.03–2.47 (4H, m), 3.65 (3H, s), 5.91 (1H, dt, *J* = 10.2, 1.8 Hz), 6.89 (1H, dt, *J* = 10.2, 4.0 Hz); ^13^C-NMR (50 MHz, CDCl_3_): δ 21.4 (CH_3_), 22.9 (CH_2_), 28.9 (CH_2_), 31.1 (CH_2_), 33.4 (CH_2_), 43.7 (C), 51.4 (CH_3_), 128.2 (CH), 148.6 (CH), 173.9 (C), 203.1 (C); MS *m/z* 196 (M)^+^, 181, 165, 136, 110, 68 (base), 55, HRMS Found *m/z* 196.1078. Calcd for C_11_H_16_O_3_ 196.1100.

### 3.3. Preparation of 6-(3-Hydroxypropyl)-6-methylcyclohex-2-en-1-ol *(**5**)*

A solution of enone **4** (172 mg, 0.88 mmol) in ether (20 mL) was treated with LiAlH_4_ (101 mg, 2.64 mmol) at 0 °C for 2 h. Usual work-up afforded the diol **5** (109 mg, 73%) after purification as a mixture of diatereomers. Oil; IR (FT): 3330, 3020, 1660 cm^−1^; ^1^H-NMR (200 MHz, CDCl_3_): δ 0.87 (3H, s), 0.88 (3H, s), 1.2–1.7 (12H, m), 1.92–2.08 (2H, m), 3.03 (2H, br s), 3.5–4.0 (6H, m), 5.5–6.0 (4H, m); ^13^C-NMR (50 MHz, CDCl_3_): δ 18.3 (CH_3_), 21.3 (CH_3_), 22.7 (CH_2_), 22.8 (CH_2_), 26.2 (CH_2_), 26.6 (CH_2_), 29.6 (CH_2_), 30.3 (CH_2_), 32.1 (CH_2_), 35.1 (CH_2_), 35.6 (C), 35.8 (C), 63.3 (CH_2_), 71.7 (CH), 72.2 (CH), 128.1 (CH), 128.6 (CH), 129.7 (CH), 129.8 (CH); MS (CI) *m/z* 169 [M-2+H]^+^, 153, 135 (base), 109; HRMS (CI) Found *m/z* 169.1223 [M-2+H]^+^. Calcd for C_10_H_17_O_2_ 169.1229.

### 3.4. Preparation of 6-(3-t-Butyldimethylsilyloxypropyl)-6-methylcyclohex-2-en-1-ol *(**6**)*

A solution of diol (**5**, 480 mg, 2.6 mmol) in CH_2_Cl_2_ (6 mL) was treated with Et_3_N (0.5 mL, 3.4 mmol) and TBDMSCl (473 mg, 3.1 mmol) at rt for 18 h. Usual work-up and purification afforded silyl ether **6** (658 mg, 82%). Oil; IR (FT): 3400, 1660 cm^−1^; ^1^H-NMR (200 MHz, CDCl_3_): δ 0.04 (6H, s), 0.88 (9H, s), 0.89 (3H, s), 1.16–1.75 (6H, m), 1.92–2.04 (2H, m), 3.58 (1H, d, *J* = 6.6 Hz), 3.60 (2H, t, *J* = 7.0 Hz), 3.74 (1H, br s), 5.62–5.74 (1H, m), 5.79 (1H, dt, *J* = 9.2, 2.6 Hz); ^13^C-NMR (50 MHz, CDCl_3_): δ −5.3 (CH_3_), 18.4 (C), 21.4 (CH_3_), 22.9 (CH_2_), 26.0 (CH_3_), 26.5 (CH_2_), 29.7 (CH_2_), 31.5 (CH_2_), 35.7 (C), 64.0 (CH_2_), 72.0 (CH), 128.3 (CH), 130.0 (CH); MS (CI) *m/z* 284 (M)^+^, 267, 227, 135 (base), 93, 83, 75; HRMS (CI) Found *m/z* 284.2155 (M)^+^. Calcd for C_16_H_32_O_2_Si 284.2172.

### 3.5. Preparation of 6-(3-t-Butyldimethylsilyloxypropyl)-6-methylcyclohex-2-en-1-one *(**7**)*

A solution of alcohol **6** (68 mg, 0.24 mmol) was oxidized with PDC (270 mg, 0.72 mmol) in CH_2_Cl_2_ in the presence of molecular sieves 3A (201 mg) at rt for 18 h. Usual work-up afforded enone **7** (59 mg, 87%) after purification. Oil; IR (FT): 1680 cm^−1^; ^1^H-NMR (200 MHz, CDCl_3_): δ 0.03 (6H, s), 0.87 (9H, s), 1.07 (3H, s), 1.32–1.63 (4H, m), 1.69–1.97 (2H, m), 2.19–2.50 (2H, m), 3.56 (2H, t, *J* = 7.0 Hz), 5.88 (1H, dt, *J* = 10.2, 2.0 Hz), 6.83 (1H, dt, *J* = 10.2, 4.0 Hz); ^13^C-NMR (50 MHz, CDCl_3_): δ −5.4 (CH_3_X2), 18.2 (C), 21.7 (CH_3_), 23.1 (CH_2_), 25.9 (CH_3_X3), 27.4 (CH_2_), 32.3 (CH_2_), 33.5 (CH_2_), 44.2 (C), 63.5 (CH_2_), 128.6 (CH), 148.5 (CH), 204.4 (C); MS (CI) *m/z* 283 [M+H]^+^, 267, 225, 151 (base); CI-HRMS Found *m/z* 283.2097 [M+H]^+^. Calcd for C_16_H_31_O_2_Si 283.2093.

### 3.6. Preparation of 4-(3-t-Butyldimethylsilyloxypropyl)-3,4-dimethylcyclohex-2-en-1-one *(**8**)*

MeLi (0.88 mL, 1 mmol) was added to a stirred solution of enone **7** (39 mg, 0.22 mmol) and the mixture was stirred at 0°C for 22 h. Usual work-up afforded a residue 36 mg), which was successively treated with PDC (140 mg, 0.36 mmol) in CH_2_Cl_2_ (4 mL) at rt for 4 h. Usual work-up and purification afforded enone **8** (33 mg, 80%). Oil; IR (FT): 1680, 1620 cm^−1^; ^1^H-NMR (200 MHz, CDCl_3_): δ 0.04 (6H, s), 0.89 (9H, s), 1.14 (3H, s), 1.18–1.76 (6H, m), 1.90 (3H, d, *J* = 1.2 Hz), 2.39 (1H, *J* = 6.4 Hz), 2.43 (1H, dd, *J* = 6.4, 2.1 Hz), 3.5–3.7 (2H, m), 5.80 (1H, d, *J* = 1.2 Hz); ^13^C-NMR (50 MHz, CDCl_3_): δ −5.3 (CH_3_X2), 18.3 (C), 20.0 (CH_3_), 24.2 (CH_3_), 25.9 (CH_3_X3), 27.7 (CH_2_), 33.4 (CH_2_), 34.1 (CH_2_), 34.8 (CH_2_), 38.2 (C), 63.2 (CH_2_), 127.2 (CH), 168.9 (C), 199.3 (C); MS (CI) *m/z* 297 [M+H]^+^, 281, 239 (base), 57, CI-HRMS Found *m/z* 297.2226. Calcd for C_17_H_33_O_2_Si 297.2250.

### 3.7. Preparation of 3-(1,2-Dimethyl-4-oxocyclohex-2-enyl)propanal *(**9**)*

Enone **8** (67 mg, 0.23 mmol) was treated with AcOH:H_2_O:THF (3:1:1) (6 mL) at rt overnight. Usual work-up afforded a residue (25 mg), which was subjected to PDC (978 mg, 2.6 mmol) oxidation in CH_2_Cl_2_ (30 mL in the presence of molecular sieves 3A (903 mg) at rt for 2 h. Usual work-up afforded enone **9** (33 mg, 80%) after purification. Oil; IR (FT): 1720, 1670, 1620 cm^−1^; ^1^H-NMR (200 MHz, CDCl_3_): δ 1.16 (3H, s), 1.65–1.97 (4H, m), 1.88 (3H, d, *J* = 1.3 Hz), 2.19–2.60 (2H, m), 2.40 (2H, t, *J* = 5.8 Hz), 5.82 (1H, d, *J* = 1.3 Hz), 9.80 (1H, t, *J* = 1.3 Hz); ^13^C-NMR (50 MHz, CDCl_3_): δ 19.9 (CH_3_), 23.8 (CH_3_), 29.8 (CH_2_), 33.3 (CH_2_), 33.9 (CH_2_), 37.7 (C), 39.1 (CH_2_), 127.9 (CH), 167.3 (C), 198.9 (C), 201.3 (CH); MS *m/z* 180 (M^+^), 162, 152, 124, 109, 95 (base), 81, 67, 55; HRMS Found *m/z* 180.1151 (M)^+^. Calcd for C_11_H_16_O_2_ 180.1151.

### 3.8. Preparation of 4-(3-Oxobutyl)-3,4-dimethylcyclohex-2-en-1-one *(**10**)*

Diol **5** (509 mg, 3 mmol) was oxidized with PDC (3.4 g, 9 mmol) in CH_2_Cl_2_ at rt overnight. Usual work-up afforded a residue (97 mg), which was treated with MeLi (9.3 mL, 10 mmol) in THF (30 mL) at rt for 16 h. The residue after usual work-up was further treated with PDC (2.6 g, 7 mmol) in CH_2_Cl_2_ at rt overnight. Usual work-up afforded enone **5** (349 mg, 60%) after purification. Oil; IR (FT): 1710, 1670, 1610 cm^−1^; ^1^H-NMR (200 MHz, CDCl_3_): δ 1.16 (3H, s), 1.64–1.97 (4H, m), 1.89 (3H, d, *J* = 1.1 Hz), 2.17 (3H, s), 2.23–2.58 (4H, m), 5 .83 (1H, d, *J* = 1.1 Hz); ^13^C-NMR (50 MHz, CDCl_3_): δ 19.9 (CH_3_), 24.0 (CH_3_), 30.1 (CH_3_), 31.6 (CH_2_), 33.3 (CH_2_), 34.0 (CH_2_), 37.8 (C), 38.5 (CH_2_), 127.7 (CH), 167.7 (C), 198.9 (C), 207.9 (C); MS *m/z* 194 (M^+^), 176, 124 (base), 109, 95, 79, 67, 55; HRMS Found *m/z* 194.1295 (M)^+^. Calcd for C_12_H_18_O_2_ 194.1307.

### 3.9. *(3*R*,3aR*,7aS*)-3-Hydroxy-3a,7a-dimethylhexahydro-1H-inden-5(6H)-one *(**11**)*)

Oil; IR (FT): 3440, 1710 cm^−1^; ^1^H-NMR (600 MHz, CDCl_3_): δ 0.90 (3H, s, 3a-CH_3_), 1.16 (3H, s, 7a-CH_3_), 1.55-1.74 (4H, m, H-1,1,2β,7β), 1.84 (1H, ddd, *J* = 15.5, 11.8, 4.9 Hz, H-7α), 2.12 (1H, dd, *J* = 14.4, 1.9 Hz, H-4β), 2.13-2.19 (1H, m, H-2α), 2.25 (1H, dtd, *J* = 14.4, 4.9, 1.9 Hz, H-6α), 2.29 (1H, dd, *J* = 14.4, 1.9 Hz, H-4α), 2.39 (1H, dddd, *J* = 14.4, 11.8, 6.0, 1.1 Hz, H-6β), 3.96 (1H, dd, *J* = 8.5, 6.6 Hz, H-3); ^13^C-NMR (150 MHz, CDCl_3_): δ 16.9 (3a-CH_3_), 22.3 (7a-CH_3_), 29.4 (C-2), 34.8 (C-1), 36.8 (C-7), 37.8 (C-6), 41.5 (C-7a), 47.5 (C-4), 50.7 (C-3a), 78.0 (C-3) , 212.5 (C-5); MS *m/z* 182 (M^+^), 164, 139, 124, 111 (base), 96, 84, 79, 69, 55; HRMS Found *m/z* 182.1298 (M)^+^. Calcd for C_11_H_18_O_2_ 182.1307.

### 3.10. *(3*S*,3aR*,7aS*)-3-Hydroxy-3a,7a-dimethylhexahydro-1H-inden-5(6H)-one *(**12**)*

Oil; IR (FT): 3320, 1710 cm^−1^; ^1^H-NMR (600 MHz, CDCl_3_): δ 0.89 (3H, s, 3a-CH_3_), 1.04 (3H, s, 7a-CH_3_), 1.53 (1H, ddd, *J* = 12.9, 10.2, 6.5 Hz, H-1β), 1.60–1.71 (2H, m, H-2α,7α), 1.91–1.95 (1H, m, H-1α), 2.07 (1H, br d, *J* = 14.3 Hz, H-4β), 2.07–2.14 (1H, m, H-7β), 2.16–2.23 (1H, m, H-2β), 2.25-2.30 (1H, m, H-6β), 2.38 (1H, br d, *J* = 14.3 Hz, H-4α), 2.37–2.42 (1H, m, H-6α), 3.96 (1H, dd, *J* = 7.7, 4.5 Hz, H-3); ^13^C-NMR (150 MHz, CDCl_3_): δ 21.6 (3a-CH_3_), 23.4 (7a-CH_3_), 30.3 (C-2), 34.9 (C-1), 35.8 (C-7), 37.6 (C-6), 41.6 (C-7a), 45.5 (C-4), 51.3 (C-3a), 81.9 (C-3), 213.0 (C-5); MS (EI) *m/z* 182 (M^+^), 164, 139, 124, 111, 95, 84 (base), 69, 55; HRMS Found *m/z* 182.1308 (M)^+^. Calcd for C_11_H_18_O_2_ 182.1307.

### 3.11. *(3*R*,3aR*,7aS*)-3-Hydroxy-3,3a,7a-trimethylhexahydro-1H-inden-5(6H)-one *(**13**)*

Oil; IR (FT): 3480, 1710 cm^−1^; ^1^H-NMR (600 MHz, CDCl_3_): δ 0.89 (3H, s, 3a-CH_3_), 1.15 (3H, s, 7a-CH_3_), 1.16 (3H, s, 3-CH_3_), 1.69 (1H, ddd, *J* = 14.2, 7.4, 4.9 Hz, H-7β), 1.77–1.81 (3H, m, H-1,2,2), 1.84 (1H, m, H-7α), 1.85–1.89 (1H, m, H-1), 1.96 (1H, dd *J* = 14.0, 1.1 Hz, H-4β), 2.20 (1H, dddd, *J* = 17.3, 9.3, 4.9, 1.1 Hz, H-6β), 2.28 (1H, d, *J* = 14.0 Hz, H-4α), 2.34 (1H, ddd, *J* = 17.3, 7.4, 4.9 Hz, H-6α); ^13^C-NMR (150 MHz, CDCl_3_): δ 16.7 (3a-CH_3_), 23.5 (3-CH_3_), 26.6 (7a-CH_3_), 36.1 (C-6), 36.3 (C-7), 37.1 (C-2), 37.6 (C-1), 42.7 (C-7a), 49.7 (C-4), 50.9 (C-3a), 84.1 (C-3), 213.7 (C-5); MS *m/z* 196 (M)^+^, 178, 150, 139, 123, 111, 84 (base), 69, 55; HRMS Found *m/z* 196.1452 (M)^+^. Calcd for C_12_H_20_O_2_ 196.1464. 

### 3.12. *(3*S*,3aR*,7aS*)-3-Hydroxy-3,3a,7a-trimethylhexahydro-1H-inden-5(6H)-one *(**14**)*

Oil; IR (FT): 3400, 1700 cm^−1^; ^1^H-NMR (200 MHz, CDCl_3_): δ 0.86 (3H, s), 0.98 (3H, s), 1.18 (3H, s), 1.40–1.50 (3H, m), 1.54-1.61 (2H, m), 1.71–1.79 (2H, m), 1.81–1.85 (1H, m), 1.88 (1H, d, *J* = 11.8 Hz), 1.88–1.94 (1H, m); ^13^C-NMR (50 MHz, CDCl_3_): δ 17.2 (CH_3_), 22.9 (CH_3_), 26.8 (CH_3_), 35.1 (CH_2_), 35.4 (CH_2_), 35.7 (CH_2_), 36.6 (CH_2_), 42.6 (CH_2_), 44.2 (C), 53.4 (C), 92.3 (C), 213.5 (C); MS *m/z* 196 (M^+^), 178, 151, 139, 125, 109, 95, 84 (base), 69, 55; HRMS Found *m/z* 196.1460 (M)^+^. Calcd for C_12_H_20_O_2_ 196.1463.

### 3.13. 8-Hydroxy-5,8,9-trimethylbicyclo[3.3.1]nonan-2-one (Mixture of Diatereoisomers) *(**15**)*

Oil; IR (FT): 3420, 1700 cm^−1^; ^1^H-NMR (200 MHz, CDCl_3_): δ 0.90 (0.25H, s), 0.93 (0.75H, d, *J* = 7.1 Hz), 0.96 (0.75H, s), 1.08 (0.25H, s), 1.40 (0.75H, s), 1.42 (0.25H, s); ^13^C-NMR (150 MHz, CDCl_3_): δ 28.0, 28.7, 31.4, 35.8, 38.2, 39.5, 40.5, 65.5, 66.6, 69.9, 71.8, 214.1 (CO); HRMS Found *m/z* 196.1465 (M)^+^. Calcd for C_12_H_20_O_2_ 196.1463.

## 4. Conclusions

We have developed methods to create *cis*-fused trimethylated hydrindanones in excellent- to high-yields by intra-molecular 5-*exo* cyclization mediated by SmI_2_ and electrolysis.
